# Oral health-related quality of life of implant-supported overdentures versus conventional complete prostheses: Retrospective study of a cohort of edentulous patients

**DOI:** 10.4317/medoral.20498

**Published:** 2015-06-02

**Authors:** Lucia Fernandez-Estevan, Eduardo J. Selva-Otaolaurruchi, Javier Montero, Fernanda Sola-Ruiz

**Affiliations:** 1 Doctor in Dentistry (DDS; PhD). Master Buccofacial Prosthetics (M.Sc). Associate Lecturer in Prosthodontics. Department of Dental Medicine. Faculty of Medicine and Dentistry. University of Valencia; 2 Doctor in Medicine (MD; PhD). Director Master Buccofacial Prosthetics. Tenured Lecturer in Prosthodontics. Department of Dental Medicine. Faculty of Medicine and Dentistry. University of Valencia; 3 Doctor in Dentistry (DDS; PhD). Tenured Lecturer in Prosthodontics. Department of Surgery. Faculty of Medicine. University of Salamanca; 4 Doctor in Medicine (MD; PhD). Adjunt Lecturer in Prosthodontics. Department of Dental Medicine. Faculty of Medicine and Dentistry. University of Valencia

## Abstract

**Background:**

This work aims to confirm if implant-supported overdentures are a good treatment option for edentulous patients and offer an improvement in quality of life compared with traditional complete prostheses (dentures).

**Material and Methods:**

This retrospective clinical descriptive study included three evaluation groups: validation group (n=57); control group of patients with complete removeable prostheses (n=56); study group of patients with implant-supported overdentures retained with the Locator® system (n=80). The study also validated the Oral Health Impact Profile-20 questionnaire. Individual protocols were created that included socio-demographic data, the Oral Health Impact Profile-20 (OHIP-20) questionnaire and Oral Satisfaction Scale (OSS). Descriptive and bivariate statistical analysis was carried out applying χ², Pearson, Kruskal-Wallis, and Student t tests, transferring data into SPSS-Windows® software from a Microsoft® Excel spreadsheet.

**Results:**

The OHIP-20 proved to be a valid instrument and provided reliable assessment of health-related quality of life among both the Spanish general population and edentulous patients. The control and study groups proved comparable, showing socio-demographic homogeneity. For patients with overdentures retained by means of the Locator® system, these restorations had significantly lower impact on quality of life (19 vs 33), both generally and for each individual questionnaire item, and much higher satisfaction with the state of their oral cavities (8.3 vs 5.3) than patients wearing dentures; both sets of data showed a direct linear relationship, so that as the level of impact on quality of life increased, perceived oral satisfaction worsened.

**Conclusions:**

Patients rehabilitated with implant supported overdentures retained by the Locator® system, presented significantly lower levels of impact on their quality of life and significantly higher oral satisfaction than patients with conventional complete prostheses.

**Key words:** Oral health-related quality of life, OHIP-20, OSS, overdentures, dental implants, complete prostheses, Locator® system.

## Introduction

Increased life expectancy in contemporary society has led to a higher percentage of edentulous patients requiring rehabilitation (1). As the McGill consensus established in 2002, overdentures, particularly mandibular implant-retained overdentures with individual attachments, are an adequate treatment for these patients (2). However, for anatomical, medical, economic, or personal reason, many patients are unable to undergo rehabilitation by these implant-retained prostheses, and so many are treated with complete removable prostheses (dentures) that often cause discomfort due to maxillary atrophy, especially in the mandible.

Removable implant-supported overdentures suppose a better quality of life in all aspects including personal comfort and even dietary habits. Overdentures produced an increased retention and stability and, most importantly, they improve the psychological response to prosthetic rehabilitation among completely edentulous patients (3). Their clinical management is extremely simple and allows the patient to maintain oral hygiene and insert and remove the prosthesis more easily than splinted implant systems. Given that this is a medium-cost treatment with reduced anatomical demands, implant-supported overdentures are the most widely used treatment option for this type of rehabilitation, although they do suffer some biomechanical limitations (2,4).

Firstly, the present study set out to revalidate the Oral Health Impact Profile-20 (OHIP-20) for use among edentulous populations and the general population in Spain, as studies of quality of life among these patients are scarce (5). Secondly – the study’s main objective – the impact on quality of life of overdentures retained by means of the Locator® system was evaluated in comparison with conventional complete prostheses (dentures), using questionnaires that measure the impact on quality of life and patients’ oral satisfaction (6,7).

The Locator® system was chosen both for its clinical advantages (reduced height, self-aligning design, anti-rotational features) and for its compatibility with a wide range of brands of dental implant in current use; nevertheless, published research on its performance is scarce.

## Material and Methods

This retrospective, descriptive clinical study was conducted in two phases with three study groups and a total sample of 193 human subjects.

- Inclusion criteria.

Validation group (n=57): patients without prostheses who attended the clinic for general oral health check-ups.

Control Group (n=56): patients with conventional complete prostheses treated at the Prosthetics deparment. They were called to a review, following the confirmation that were carriers of this treatment.

Study Group (n=80): patients rehabilitated by implant-supported removable overdentures retained with the Locator® system (Zest Anchor, Escondido USA) treated at the Prosthetics deparment. They were called to a review, following the confirmation that were carriers of this treatment.

It took place at the Dental Clinic in the Department of Dental Medicine of the University of Valencia Faculty of Medicine and Dentistry.

The first phase set out to confirm the validity of the cultural and linguistic adaptation of the OHIP-20 to Spanish populations (5). The second phase studied the impact on oral quality of life and patient satisfaction.

The study protocol was approved by the University of Valencia Human Research Ethics Committee (Additional file 1). All patients were informed of the study procedure and gave their signed consent to take part; the study was designed to ensure that all current ethical and legal requirements would be met.

A two-step bibliography search was performed in the Pubmed® Medline database using the search terms “oral” and “health.” The search results were then refined applying the terms “quality”, “life” and “edentulous,” reviewing the literature identified, as well as other published researched referenced therein.

The study used the reduced version of the OHIP, specially adapted for edentulous patients – the OHIP-EDENT – consisting of 20 questions (6). It includes conceptual domains such as functional limitation, pain, psychological discomfort physical disability, psychological disability, social disability and handicap. The responses to each item take the form of a Likert scale: 0-never; 1-hardly ever; 2-occasionally; 3-quite often; 4-very often. The total score of the impact on oral health-related quality of life was calculated by the sum of the scores obtained in 20 items, thus, the higher the score, the higher the frequency of impact is, being 80 the maximum impact score. The scale is easy to use and minimizes the influence of the researcher on the patients’ responses, as patients can fill out the questionnaire independently. This questionnaire is simple and everyone can answer or understand it, if anyone had any difficulty the auxiliary personnel could help them, avoiding the influence of the researcher.

Having chosen to work with the OHIP-20, it seemed appropriate to re-validate its use for the target population, using the version linguistically and culturally adapted to Spanish populations (OHIP-20Esp) from the original English language version (5,6). For this reason, the Spanish OHIP-20Esp was applied to a population independent of the main study groups, validation group (n=57), as well as patients wearing removable complete prostheses (control group, n=56), who also responded to a question about their understanding of the questionnaire. These groups provided Baseline Oral Impact Data, which was later applied to the group of patients rehabilitated with Locator® system-retained overdentures (Study Group, n=80).

The Oral Satisfaction Scale (OSS) was also used, a visual analogue scale (VAS) with scores of 0-10, which is quick and easy to use, an ideal accompaniment to the OHIP-20Esp (6).

All socio-demographic data were collected in individual evaluation protocols, which patients filled out under supervision. Each patient filled out the OHIP-20Esp questionnaire and OSS VAS independently in order to avoid any possible bias arising from the interference of the researcher. The data obtained was analyzed, calculating the sum total of all responses to provide an overall result and also the sum totals for each domain in order to identify the areas that had the most impact on oral quality of life. The internal coherence of the questionnaire was also assessed.

Cronbach’s α was used to confirm the validity, reliability and consistency of the OHIP-20Esp as an instrument for measuring patients’ oral quality of life.

Statistical analysis was performed using SPSS-Windows® software (Statistical Package for the Social Sciences. SPSS Inc. Chicago, Illinois, USA), importing data from a single Microsoft® Excel spreadsheet, which included all data obtained in the socio-demographic evaluation protocol, OHIP-20Esp and OSS. Descriptive and bivariate analyses were performed, applying the Pearson χ², Kruskal-Wallis, Mann-Whitney and Student t tests. The significance level established for all bivariate analysis was 5%, any p-value below 0.05 indicating a statistically significant difference.

## Results

- Validation Group (n=57).

The typical patient profile was a 54-year-old woman who cleans her teeth once or twice a day and visits the dentist once a year, the previous dental appointment having taken place about six months earlier (although 30% of subjects were unable to remember the date of the previous visit to the dentist). The main motive for the appointment was because some oral health problem had arisen (50.9%) - only 33.3% of subjects were found to undergo regular check-ups. 35.1% of subjects had received basic school education; 63.2% did not work. Most of the group were nonsmokers (72%); 31.6% did not present any pathology and 28.1% suffered pathologies classed as ‘other’, among which depression and sleep disorders were prevalent; 44% did not take any medication. It is worth mentioning that 16% suffered some sort of disorder and had been prescribed medication but were unable to remember the name of the medication or the reason it had been administered.

This group responded to an item on how well they understood the OHIP-20Esp; 89.5% of subjects stated that they understood properly the full questionnaire, a datum that confirms the apparent validity of its cultural and linguistic adaptation to Spanish. Furthermore the Cronbach’s α value (0.92) supported the internal consistency of the questionnaire.

Oral quality of life data: OHIP-20Esp and OSS.

When impact on oral quality of life was assessed with the OHIP-20Esp, the validation group produced low scores, the mean score being 18.4 out of 80. The level of satisfaction obtained in the OSS was low-to-moderate with a mean of 5.2 out of 10. Spearman’s correlation coefficient was used to analyze OHIP-20Esp and OSS outcomes together, measuring the correlation between two ordinal variables; this was -0.36 following a linear negative correlation with a significance of over 99%. As OHIP-20Esp scores increase (worse quality of life) OSS results decrease (worse oral satisfaction) and vice-versa.

- Control Group: patients with mandibular conventional complete removable prostheses (n=56).

The typical socio-demographic profile of this patient population was a 70-year-old woman, who cleans her teeth once or twice a day (58.9%) and makes one or more visits to the dentist per year (48.2%). However, a high proportion of this group (25%) only saw the dentist once in three years. The most frequent motives for dental appointments were for a general check-up (46.4%) or in response to a particular problem (51.8%). The majority (71.4%) had received little or no education and more than half were retired (58.9%), with 83.9% of subjects not in work. 67.9% were nonsmokers, 14.3% were diabetic, 23.2% suffered from osteoporosis, and 33.9% from cardiovascular disease. Many subjects (44.6%) did not know what medication they were taking. For this group, the study also noted the state of the antagonist arch and 89.3% had completely rehabilitated upper maxillas. ( Table 1, Table 2).

**Table 1:**
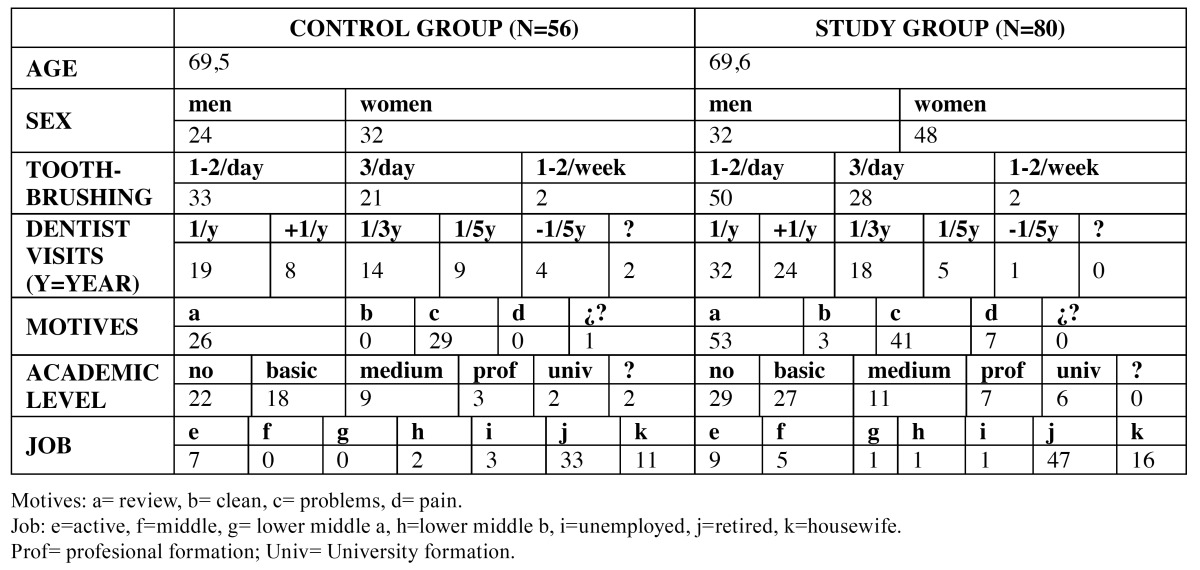
Sociodemographic data control and study group.

**Table 2:**
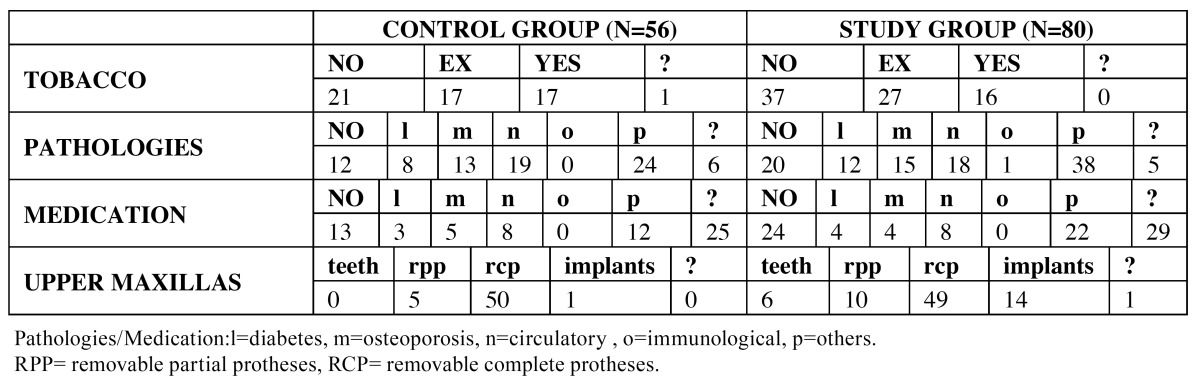
Sociodemographic data control and study group (2).

This group also responded to a question about their understanding of the OHIP-20Esp questionnaire; 92.8% stated that they understood perfectly all the items questionnaire, supporting the face validity. Furthermore the Cronbach’s α value (0.92) supported the internal consistency of the questionnaire.

Oral quality of life: OHIP-20Esp and OSS.

This group scored 33 out of 80 in the OHIP-20Esp, indicating a middle-range quality of life. When domains were analyzed individually, the items having the most impact were as follows: OHIP-20.13 “Have you had to interrupt meals because of problems with your teeth, mouth or dentures?” (Mean score 2.8 out of a maximum score of 4), OHIP-20.10 “Have you had to avoid eating some foods because of problems with your teeth, mouth or dentures?” (Mean score 2.6) and OHIP-20.2 “Have you had food catching in your teeth or dentures?” (Mean 2.6).

The mean OSS score for this group was 5.3 out of 10, indicating low-to-medium oral satisfaction.

**Figure 1:**
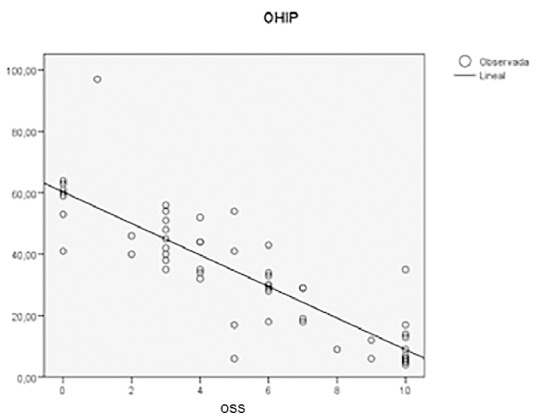
Scatter plot between OHIP-20Esp VS OSS among the control group.

Analyzing OHIP-20Esp and OSS outcomes together, Pearson’s correlation coefficient, used for normal variables was -0.85, following a linear negative correlation with a significance of over 99%. As OHIP-20Esp scores increase (worse quality of life) OSS results decrease (worse oral satisfaction) and vice-versa. (Fig. 1).

The control group showed no statistically significant relations between socio-demographic data and the OHIP and OSS scores. Nevertheless, subjects who had received a university education and were in work reported greater impact on quality of life and lower oral satisfaction, although without statistical significance.

- Study group: Patients with implant-supported Locator®-retained mandibular overdentures (n=80).

The typical socio-demographic profile in this group was a 70-year-old woman who cleans her teeth once or twice a day (62.5%) and visits the dentist one or more times per year for a check-up or in response to some particular problem. Most subjects (70.1%) had received little or no education and more than half were retired (58.8%), with 80% not in work. 80% were nonsmokers, 15% diabetics, 19% suffered osteoporosis, and 22.5% cardiovascular disease. Most subjects did not know what medication they were taking and 30% did not need any kind of medical treatment. For this group, the study also noted the state of the antagonist arch; 61.3% of subjects had completely rehabilitated upper maxillas. ( Table 1, Table 2).

Oral quality of life: OHIP-20Esp and OSS.

Patients with mandibular implant-supported overdentures retained with the Locator® system, had an overall OHIP-20Esp score of 19 out of 80, indicating good quality of life and low level of impact. When the different domains were analyzed individually, the items with most impact were as follows: OHIP-20.2 “Have you had food catching in your teeth or dentures?” (Mean score 1.9 out of 4), OHIP-20.1 “Have you had difficulty chewing any foods because of problems with your teeth, mouth, or dentures?” (Mean score 1.6), OHIP-20.8 “Have you been worried by dental problems?” (Mean score 1.5).The OSS obtained s mean score of 8.3 out of ten for this group, indicating a high level of satisfaction with oral health.

**Figure 2:**
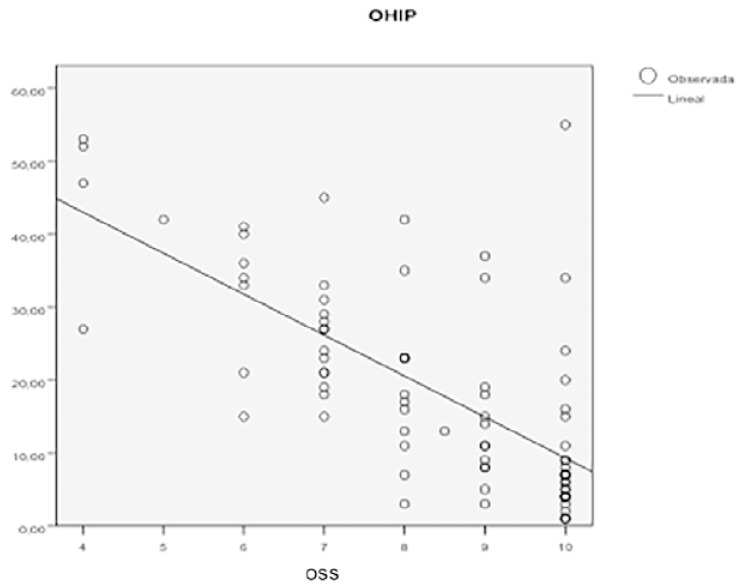
Scatter plot between OHIP-20Esp VS OSS among the study group.

Analyzing OHIP-20Esp and OSS outcomes together, the Pearson correlation coefficient was -0.7, following a linear negative correlation with a significance of over 99%. As OHIP-20Esp scores increase (worse quality of life) OSS results decrease (worse oral satisfaction) and vice-versa. (Fig. 2).

The study also analyzed possible relations between socio-demographic data and OHIP-20Esp and OSS scores. It was found that only age showed statistical significance (*p*<0.05) in relation to OHIP-20Esp scores and the frequency of visits to the dentist showed significance in relation to both the OHIP-20Esp and OSS scores. Elderly patients gave lower scores in the OHIP-20Esp, indicating lower levels of impact on oral quality of life, which may suggest that these patients have lower expectations, and do not demand total rehabilitation of the oral functions and esthetics that they have lost. Patients who make more frequent visits to the dentist were found to be more demanding, to expect more from their prostheses, and so gave higher OHIP-20Esp scores, indicating higher levels of impact on oral quality of life, and lower OSS scores indicating lower levels of satisfaction. Gender, employment/unemployment, and educational level showed no statistical significance, although the study population was highly homogeneous.

- Comparison of study and control groups.

The study identified points of comparison and difference between the groups of subjects with overdentures (study group) and conventional dentures (control group). The first step was to eliminate the possibility that the domains that showed differences might be due to socio-demographic conditions. Statistically significant differences were only found between study group patients who made more frequent visits to the dentist than the control group. The typical patient in both groups was a woman of 70 years of age of low educational level who does not work; both groups showed similar oral hygiene habits. ( Table 1, Table 2).

The study group gave a mean overall OHIP-20Esp score of 19 out of 80 compared to 33 in the control group. In this way, it can be affirmed that patients with mandibular implant-supported overdentures retained with the Locator® system gave significantly lower scores (*p*<0.001 Student t-test), showing lower levels of impact on oral quality of life than patients rehabilitated with conventional dentures. When OHIP-20 domains were analyzed individually, three out of the five items awarded the highest scores by these groups coincide. In this way, these were perceived as the areas that most influenced oral quality of life: OHIP-20.2 food catching in dentures, OHIP-20.10 avoiding some foods and OHIP-20.5 discomfort experienced at some moment. Coincidences were also found in the domains that received the lowest scores, showing that these - all items related to social disability – had the least impact as far as these subject groups were concerned.

Study group subjects gave a mean OSS score of 8.3 and control group subjects 5.3. In this way, patients with mandibular implant-supported overdentures retained with the Locator® system were significantly more satisfied (*p*<0.001, Student t-test) with the state of their mouths than subjects wearing conventional complete prostheses.

## Discussion

Although with time, prevention may eventually reduce edentulism, the percentage of patients without teeth is increasing due to longer life expectancy, so that edentulous patients will continue to be a relevant proportion of dental patients for the foreseeable future (8). Undoubtedly, edentulism is considered a negative condition that compromises patient quality of life (9-12). Implant-supported overdentures offer the dentist an opportunity to bring about improvement to both the oral health and quality of life of these patients.

Clinical indicators by themselves fail to identify the limitations patients suffer when carrying out everyday activities (13). For this reason, quality of life assessment (8) has become an important factor in the evaluation of treatment outcomes (5,14,15) and one of the most significant factors for evaluating treatment success for dental implants supporting overdentures (8). A topic of only recent interest, the first study related to oral quality of life among adults in Spain was published in 2008 (16). Although no gold standard for measuring quality of life has been established, a range of instruments have been designed to make objective evaluations oral quality of life (13,17,18). In 1994, Drs. Slade and Spencer developed and tested the Oral Health Impact Profile (OHIP-49) and this questionnaire has been used by various researchers (14,19-21) for evaluating patients rehabilitated with conventional or implant-retained removable prostheses, both in its original version (OHIP-49) and in reduced versions aimed at this specific patient group (OHIP-EDENT, OHIP-20) (8,22). Although other questionnaires are recognized internationally (7,13,16,23), none have turned out to be as effective and as well-focused on edentulous patients as the OHIP-20. Nevertheless, although it measures the impact of rehabilitation treatments on oral quality of life in terms of frequency, it fails to assess the severity of the different domains analyzed, a failing that may represent a considerable bias in its assessment of quality of life and its chief limitation (18). The minimum sample size for differentiating between groups when using the OHIP-20 seems to be about 50 patients (5,10); in the present case, the study group included 80 subjects and the control group 56.

The method used in the present study to validate the adaptation of this international questionnaire for use among Spanish populations was similar to others described in previous studies (5,13,16), which showed the OHIP-20 to be a valid and reliable instrument for assessing quality of life among edentulous patients (5) and for comparing rehabilitation techniques. The statistical tests applied to validate comparisons between distributions identified high statistical power, ranging from 75% in the worst case to 95% in the best. It was also necessary to test the internal validity of the OHIP-20Esp’s measuring scale. For the 20 items that make up the questionnaire, Cronbach’s α was 0.92, a high value that confirms the validity, reliability and consistency of the OHIP-20Esp as an instrument for measuring patients’ oral quality of life.

The visual analogue oral satisfaction scale (OSS), a unidimensional scale, has been shown to be a simple but discerning indicator of oral well-being, and can be used for measuring oral health-related quality of life (16). Applied together with a multidimensional questionnaire-based measuring instrument (such as the OHIP-20), it provides a complimentary perspective on patients’ perception of their oral health (16).

An extensive range of literature has evaluated quality of life among edentulous patients rehabilitated with implant-supported overdentures compared with conventional complete prostheses (dentures) (12,14,18,19). But very few studies have used the Locator® retention system, and even fewer have compared this with a control group of denture-wearing patients (24).

It may be stated that patients experience an improvement in function and in their overall self-image after receiving im-plant-supported rehabilitation – regardless of whether this is removable or fixed and regardless of the type of retention used for removable overdentures – a fact that contradicts the widespread belief that patients prefer fixed rehabilitations. Overdentures not only suppose improved retention and masticatory efficacy, but also boost patient confidence, and so improve their social life (8,11,12,19,25,26). However, some authors differ from the present study in their findings, and have not found differences in quality of life between patients rehabilitated with mandibular overdentures and those with conventional complete prostheses (14,21).

Any disorder that has an impact on patients’ well-being is influenced by socio-demographic, social, psychological and environmental factors, variables that modulate individual perception of oral quality of life (16). Patients rehabilitated with implant-supported overdentures require a higher number of dental appointments and longer treatment time than patients rehabilitated by conventional means, a situation that might be considered a disadvantage. The present study observed that subjects who visited the dentist more often had higher expectations and made higher demands of their prostheses, which then had a greater impact on quality of life. Women generally give lower scores for satisfaction than men, particularly in relation to the aesthetic outcomes of rehabilitation (9,10,16,27,28). Some authors state that satisfaction increases with age and that tolerance of complications increases, while others consider that older patients display lower satisfaction as more problems occur. The present study observed that elderly patients perceived lower levels of impact on oral quality of life, while gender, employment/unemployment, educational level and job status had no statistical significance within this homogeneous study population.

In one study using the OHIP-20, patients with overdentures retained by means of the Locator® system obtained a mean total of 9.6 ± 12.6 compared with 17.3 ± 8.6 for a control group of patients with conventional complete prostheses (5). The present work obtained an overall OHIP-20 score of 18.96 out of 80 among patients with Locator®-retained overdentures, compared with 33 out of 80 for a control group, indicating that these rehabilitations had a low impact on oral quality of life. The OSS found a high level of satisfaction, with a mean overall score of 8.3 out of 10.

Minimal important difference (MID) (29) is “The smallest difference in score in the domain of interest which patients perceive as beneficial and which would mandate, in the absence of troublesome side-effects and excessive cost, a change in the patient’s management” (17,29). MID for the OHIP-20 ranges between 7 and 10, varying between different populations and clinical contexts; for this reason, it is appropriate to estimate MID according to each particular case (17). In the present study, MID was 14 points between the two groups, so that it can be extrapolated that there are differences between treatment groups and that overdentures are the better therapeutic option.

## Conclusions

1 The OHIP-20Esp is a valid and reliable instrument for measuring oral quality of life of edentulous patients among the Spanish population.

2 Implant-supported Locator®-retained mandibular overdentures had low impact on patient quality of life, as measured by means of the Spanish version of the Oral Health Impact Profile 20 (OHIP-20Esp), which obtained a mean score of 19 out of 80, indicating good quality of life. Patients with overdentures presented significantly lower levels of impact on their quality of life, both overall and for each questionnaire domain, than patients rehabilitated with conventional complete removable prostheses (33 out of 80).

3 The degree of oral satisfaction of patients rehabilitated with overdentures, measured by means of the Oral Satisfaction Scale (OSS) was high (8.3 out of 10), showing that this patient group was satisfied with the treatment received. This satisfaction was significantly higher than patients with complete prostheses (5,3 out of 10).
